# A simple, time- and cost-effective, high-throughput depletion strategy for deep plasma proteomics

**DOI:** 10.1126/sciadv.adf9717

**Published:** 2023-03-29

**Authors:** Arthur Viode, Patrick van Zalm, Kinga K. Smolen, Benoit Fatou, David Stevenson, Meenakshi Jha, Ofer Levy, Judith Steen, Hanno Steen, On behalf of the IMPACC Network

**Affiliations:** ^1^ Department of Pathology, Boston Children’s Hospital, Boston, MA, USA.; ^2^ Harvard Medical School, Boston, MA, USA.; ^3^ Department of Neuropsychology and Psychopharmacology, EURON, Faculty of Psychology and Neuroscience, Maastricht University, Maastricht, Netherlands.; ^4^ Precision Vaccines Program, Division of Infectious Diseases, Boston Children’s Hospital, Boston, MA, USA.; ^5^ Broad Institute of MIT and Harvard, Cambridge, MA, USA.; ^6^ F. M. Kirby Neurobiology Center, Boston Children's Hospital, Boston, MA, USA.

## Abstract

We introduce a cost-effective, robust high-throughput–compatible plasma depletion method enabling in-depth profiling of plasma that detects >1300 proteins per run with a throughput of 60 samples per day. The method has been fully validated by processing >3000 samples with no apparent batch effect at a cost for the depletion step of ~$2.5 per sample.

## Teaser

A simple plasma protein precipitation method is described to overcome the challenges of plasma proteomics.

## INTRODUCTION

The importance of plasma as a source of potential biomarkers cannot be overstated; as the most collected biofluid globally, it is used for ~40% of clinical tests administered. Given its importance, the collection of plasma is routine, robust, and performed on thousands of subjects in clinics and drug trials every day. As such, plasma has become the focus of many academic, pharmacological, biomedical, and clinical pursuits as it is an invaluable source of biomarkers. However, identifying protein biomarkers in plasma has been limited because the most abundant 22 proteins in plasma account for 99% of all protein content, preventing the detection of less abundant proteins using discovery mass spectrometry (MS)–based proteomics. Thus, only the most abundant ~300 proteins, aka “classical plasma proteins” (*1*), are reliably detected across most neat plasma proteomics studies.

Studies of clinically relevant biomarkers in human translational research benefit from high-throughput (HTP) proteomic methodologies [e.g., >50 samples per day (SPD)] to analyze cohorts with hundreds or thousands of samples (*2*, *3*). Thus, extensive fractionation and costly antibody-based depletion methods to map plasma proteins beyond the classical plasma proteome are not viable options: Extensive fractionation severely affects throughput with the use of the liquid chromatography (LC)/MS system, while antibody-based depletion methods are expensive and not sufficiently robust for biological samples with variable pH and/or salt concentrations. Furthermore, these methods are laborious with complex multistep protocols.

To be useful for clinically relevant biomarker discovery studies, a pipeline must meet the following requirements: (i) have HTP capabilities, i.e., enable processing and analysis of large numbers of plasma samples; (ii) be cost-efficient; (iii) be time-efficient; (iv) display minimal batch effects; and (v) enable reliable detection of plasma proteins at lower concentrations than the classical plasma proteome. These less abundant proteins are primarily tissue-derived, show pathology-specific abundance differences, and likely represent biomarker candidates for the early detection of diseases affecting a wide range of organs.

## RESULTS AND DISCUSSION

**Fig. 1.**
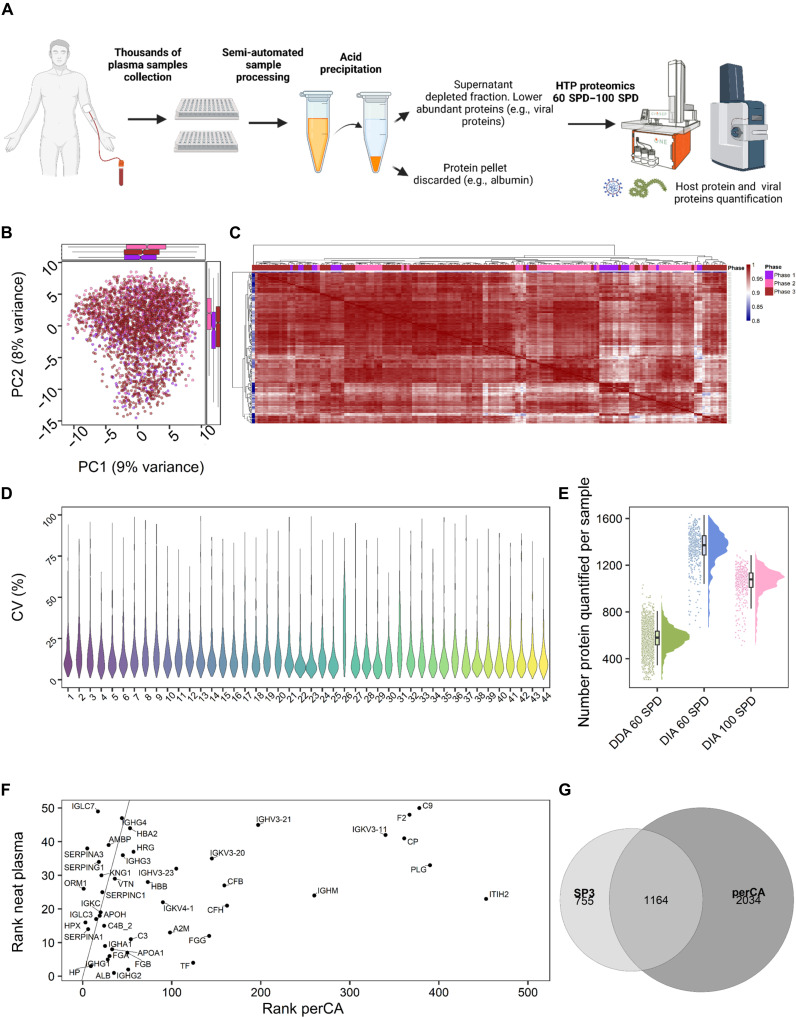
Perchloric acid depletion workflow and method validation. (**A**) Workflow: PerCA plasma proteomic platform. (**B**) Principal components analysis to identify possible batch effects between phases 1 and 3 (colored in purple, pink, and brown) acquired over a 10-month period. (**C**) Sample-to-sample correlation of all reference plasma samples from the 44 plates. (**D**) Four reference plasma samples were analyzed on each of the 44 plates enabling calculation of the coefficient of variation (CV) for each protein within each plate. The overall average CV: 15.7% with one plate at 23%. (**E**) Raincloud plots of protein quantified per sample for data-dependent acquisition (DDA) 60 SPD, data-independent acquisition (DIA) 60 SPD, and DIA 100 SPD: on average, 576, 1343, and 1051 proteins, respectively. (**F**) Comparison of the ranks of the 50 most abundant plasma proteins before (*y* axis) and after perCA depletion (*x* axis). The line corresponds to the *x* = *y* line indicating proteins enriched (left of the line) and depleted (right of the line) by perCA. (**G**) Venn diagram comparison of proteins identifiable in neat plasma (1919) and after perCA depletion (3198). Created with BioRender.com

Here, we introduce an HTP-compatible and reproducible biochemical plasma depletion method that meets all the requirements listed above. The method is based on selective protein precipitation by perchloric acid (perCA) (*4*). Our implementation of the method, which is built upon methods used for the enrichment of, e.g., histone, high-mobility protein, and/or peptides (*5*–*7*), is depicted in Fig. 1A. Briefly, (i) the serum or plasma (irrespective of coagulant) sample is collected and (ii) 50 μl is treated with perCA to a final concentration of 3.5% before the centrifugation at a *g*-force compatible with multititer plates—a prerequisite for reproducible HTP processing. (iii) Using a liquid handling robot, the supernatant is removed from the pellet and transferred to the solid-phase extraction (SPE) plate to remove the acid and rebuffer. (iv) The proteins are eluted and trypsinized before loading onto the Evosep tips for LC/MS analysis on a timsTOF Pro 2 mass spectrometer (Bruker Daltonics; Billerica, MA) equipped with an Evosep One LC system (Evosep, Odense, DK). (v) The acquired data are analyzed effectively through computational parallelization (*8*).

The robustness of our method was tested on ~3200 plasma samples from the National Institute of Allergy and Infectious Diseases (NIAID)–funded IMmunoPhenotyping Assessment in a COVID-19 Cohort (IMPACC) study (*9*, *10*), which enrolled 1141 hospitalized patients with coronavirus disease 2019 (COVID-19) between May 2020 and March 2021 collecting up to six longitudinal samples per participant during hospitalization. The samples from the hospitalization period were shipped and processed in 44 plates assigned to three different batches over a 10-month period: December 2020 (*n* = 437), April 2021 (*n* = 1047), and September 2021 (*n* = 1715). In addition to the samples, each plate also contained four reference plasma samples that enabled the assessment of the reproducibility and robustness of our method across this extended period. Principal components analysis (Fig. 1B), correlation of all the reference plasma samples from the 44 plates (Fig. 1C), and the observed SDs (Fig. 1D) highlighted excellent precision and suggested minimal, if any, batch effects. The absence of batch effects possibly results from the extreme conditions during protein depletion, which makes it refractory to differences in, e.g., salt concentration and/or pH, or general sample processing; these parameters are known to affect the specificity and affinity of antibodies. In addition to the outstanding robustness, the perCA depletion has the competitive advantage of being substantially cheaper than other existing methods: The depleting reagent, i.e., perCA, costs <3 cents per sample, with the only additional costs coming from SPE plates [e.g., μSPE hydrophilic-lipophilic balance (HLB) plates] for desalting/rebuffering, although this cost can be reduced when cleaning and reusing the plates. As such, the costs of our depletion strategy are ~1/100 when compared to other commercial solutions or services, enabling greater access to reproducible plasma protein depletion.

The extent of the depletion is readily assessed by comparing the ranks of the 50 most abundant classical plasma proteins before and after depletion (Fig. 1F). For example, albumin, the most abundant plasma protein that accounts for ~55% of the total plasma protein (*1*), drops after perCA depletion to rank 35. Similarly, after perCA depletion, *IGH2* and *ITIH2* dropped in rank from 2 to 51 and from 23 to 453, respectively.

To assess the perCA depletion further, we analyzed all 3199 IMPACC samples in data-dependent acquisition (DDA) mode. These DDA data were used to generate a spectral library for efficiently searching data-independent acquisition (DIA) data. The resulting library comprised 25,803 peptides derived from 3196 proteins. This spectral library enabled the identification of an average of 1343 proteins per run at a throughput of 60 SPD in DIA. The average number of detected plasma proteins remained above 1000 even at a throughput of 100 SPD (Fig. 1E). For comparison, we also generated a spectral library from ~1500 nondepleted neat plasma samples, composed of 19,148 peptides associated with 1920 proteins. The Venn diagram demonstrates that perCA doubles the number of detectable proteins compared to neat plasma (Fig. 1G).

**Fig. 2.**
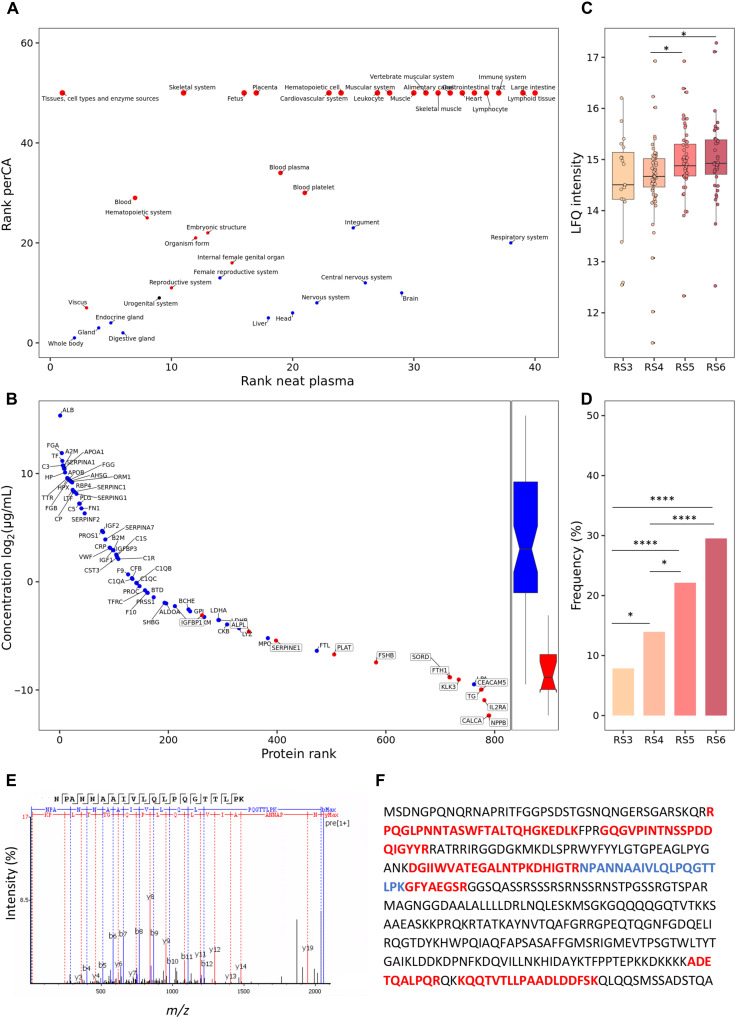
Optimization through perchloric acid depletion enables unique plasma protein detection including biomarkers and SARS-CoV-2 nucleoprotein. (**A**) Tissue enrichment analysis before and after perCA depletion: Top 40 enriched tissues after the depletion relative to rank before depletion. Color and size indicate depletion/enrichment and fold change. (**B**) Seventy-three percent (77 of 106) of all Food and Drug Administration–approved biomarkers were identified: 59 without depletion (blue) and 18 uniquely after depletion (red), all of which have a markedly lower concentration range: 0.19 to 117 ng/ml versus 1.4 to 42 × 10^6^ ng/ml. The *y* axis displays the log_2_ of the protein concentration in micrograms per milliliter. (**C**) The abundance [label-free quantitation (LFQ) intensity, arbitrary units] of severe acute respiratory syndrome coronavirus 2 (SARS-CoV-2)–derived nucleoprotein (NP) at admission to the hospital for each respiratory status (RS): Marginally significant (*t* test) differences only between RS4/RS5 (*P* = 0.023) and RS4/RS6 (*P* = 0.036). (**D**) Frequency of SARS-CoV-2–derived NP at admission to the hospital for each RS: Frequency NP: Statistically highly significant (Fisher’s exact test) between RS3/RS4 (*P* = 3.8 × 10^−2^), RS3/RS5 (*P* = 8.1 × 10^−5^), RS3/RS6 (*P* = 4.6 × 10^−7^), RS4/RS5 (*P* = 2.2 × 10^−2^), and RS4/RS6 (*P* = 3.1 × 10^−4^). (**E**) Tandem mass spectrum of the unique NP-derived peptide NPANNAAIVLQLPQGTTLPK^2+^ [mass/charge ratio (*m/z*) = 1030.579] using PEAKS Studio 10.5. (**F**) Ten unique peptides cover 29% of the NP sequence. Created with BioRender.com

Using both libraries, we compared the tissue origin of the detectable proteomes (https://tissues.jensenlab.org/) (Fig. 2A) before and after depletion. The perCA depletion reveals several proteins associated with a wide range of organs/tissues (e.g., skeletal system, cardiovascular system, muscular system, gastrointestinal tract, lymphoid tissue, and large intestine) (see tables S1 and S2 for a complete list), which are either not or barely detectable in neat plasma.

Our study reveals the utility of the perCA workflow compatible with plasma as a systemic biofluid, which reflects the status of multiple organs and tissues. The improved analytical depth of the perCA approach is evident in considering Food and Drug Administration (FDA)–approved biomarkers. Of the currently FDA-approved 106 biomarkers (*11*), 59 proteins can be detected in both neat and perCA-depleted plasma covering a concentration range from 42 mg/ml to 1.4 ng/ml (*11*). However, the perCA protocol enables the detection of an additional 18 FDA-approved biomarkers, covering a markedly lower concentration range of 117 ng/ml to 190 pg/ml (Fig. 2B and table S3). The perCA fraction featured six additional FDA-approved biomarkers that were not considered bona fide plasma proteins when the Plasma Proteome Database was created (*12*, *13*).

The true potential of the perCA depletion–based plasma proteomics pipeline is illustrated through the analysis of the IMPACC study. The perCA-depleted plasma enabled the first untargeted detection of severe acute respiratory syndrome coronavirus 2 (SARS-CoV-2)–derived proteins in COVID-19 patient plasma analyzed by shotgun discovery plasma proteomics (Fig. 2, C and D). We identified the SARS-CoV-2 nucleoprotein (NP) in ~10% of the samples. The ten NP-derived peptides (e.g., NPANNAAIVLQLPQGTTLPK; see Fig. 2F) cover 29% of the NP sequence (Fig. 2D and table S4), identifying the presence of viral proteins in blood, although SARS-CoV-2 is not a blood-borne pathogen.

To assess whether the observation of the viral NP protein is associated with disease severity, we correlated NP protein abundance and NP observation frequency with respiratory status (RS) at hospital admission (*9*, *10*). RS3 is the least severe status—hospitalization without oxygen therapy—while RS6 is the most severe, indicating the need for invasive mechanical ventilation [e.g., extracorporeal membrane oxygenation (ECMO)]. Participants with increasingly severe RS status demonstrated only a small increase in NP protein abundance (Fig. 2E). In contrast, a clear and significant increase in the observation frequency of NP is detected. The percent NP positivity correlated with COVID-19 severity: Only ~8% of the RS3 patients’ plasma was NP-positive, whereas percent NP positivity increased to 14, 22, and 30% for RS4 to RS6, respectively (Fig. 2F and tables S5 and S6). This finding is consistent with the observation that blood viremia (measured by viral RNA sequencing) is associated with severe COVID-19 (*14*).

In summary, we present a validated, time- and cost-efficient, HTP protocol for depletion of the most abundant plasma proteins, which reveals many tissue- and organ-specific proteins of lesser abundance. In contrast to existing depletion protocols, the perCA depletion is cost-efficient (2 to 3 cents per sample for the precipitation reagent), insensitive to a wide range of starting conditions normally encountered in body fluids, and extremely robust and reproducible as demonstrated in processing and analyzing >3000 samples from the IMPACC study. The detection of viral proteins in plasma in discovery mode and the detection of >1000 plasma proteins per sample at a throughput of 100 SPD highlight the power and sensitivity of this perCA depletion–based plasma proteomics pipeline.

## MATERIALS AND METHODS

### Respiratory status

As recently reported by the IMPACC team (*10*), COVID-19 severity was assessed using a seven-point ordinal scale (OS) for RS, adapted from the World Health Organization COVID-19 and NIAID disease ordinal severity scales. The seven-point OS includes the following: OS1 = not hospitalized, no limitations; OS2 = not hospitalized, activity limitations, or requires home O_2_; OS3 = hospitalized, not requiring supplemental O_2_; OS4 = hospitalized, requiring O_2_; OS5 = hospitalized on noninvasive ventilation or high-flow O_2_; OS6 = hospitalized on invasive mechanical ventilation and/or ECMO; OS7 = death.

### Reference plasma samples

Plasma from 18 healthy individuals was collected. Equal volumes of these plasma samples were pooled, aliquoted, and frozen for future use.

### Ethics statement

Ethics NIAID staff conferred with the Department of Health and Human Services Office for Human Research Protections (OHRP) regarding the potential applicability of the public health surveillance exception [45CFR46.102 (*9*, *15*)] to the IMPACC study protocol. OHRP concurred that the study satisfied the criteria for the public health surveillance exception, and the IMPACC study team sent the study protocol, participant information sheet for review, and assessment to institutional review boards (IRBs) at participating institutions. Twelve institutions elected to conduct the study as public health surveillance, while three sites with prior IRB-approved biobanking protocols elected to integrate and conduct IMPACC under their institutional protocols (University of Texas at Austin, IRB 2020-04-0117; University of California San Francisco, IRB 20-30497; Case Western reserve university, IRB STUDY20200573) with informed consent requirements. Participants enrolled under the public health surveillance exclusion were provided information sheets describing the study, samples to be collected, and plans for data deidentification and use. Those who requested not to participate after reviewing the information sheet were not enrolled. In addition, participants did not receive compensation for study participation while inpatient and subsequently were offered compensation during outpatient follow-ups (*10*).

### Sample preparation

Fifty microliters of neat plasma samples was diluted with 450 μl of water and 25 μl of perCA was added. After vigorous agitation, the suspension is kept at −20°C for 15 min. The suspension is then centrifuged for 60 min (4°C, 3200*g*) and the supernatant is kept. The supernatant is then mixed with 40 μl of 1% trifluoroacetic acid and loaded onto a μSPE HLB plate, previously conditioned with 300 μl of methanol and two times with 500 μl of 0.1% trifluoroacetic acid. Proteins were eluted from the μSPE HLB plate with 100 μl of 90% acetonitrile with 0.1% trifluoroacetic acid. After elution, the samples were dried with a Speedvac. The samples were resuspended with 35 μl of 50 mM ammonium bicarbonate and digested with 10 μl trypsin (Promega, 500 ng) overnight at 37°C. Digestion is stopped by the addition of 5 μl of 10% formic acid. The samples were stored at −80°C before LC/MS analysis.

### Sample acquisition DDA

Two microliters of tryptic peptides was loaded onto Evotip and analyzed using an Evosep One LC (EVOSEP) connected to a timsTOF Pro (Bruker). We used a C18 column 8 cm by 150 μm (1.5-μm particle size) from PepSep. The Evosep One method was 60 SPD (21-min gradient, cycle time of 24 min), and the mass spectrometer was operated in DDA-Parallel Accumulation-Serial Fragmentation (PASEF) mode. Four PASEF MS/MS scans were triggered per cycle. DDA-PASEF parameters included the following: mass/charge ratio (*m/z*) range: 100 to 1700, mobility (1/K0) range: 0.70 to 1.45 V⋅s/cm^2^, the accumulation and ramp time were 100 ms. Target intensity per individual PASEF precursor was set to 5000. The values for mobility-dependent collision energy ramping were set to 51 eV at an inversed reduced mobility (1/K0) of 1.45 V·s/cm^2^ and 21 eV at 0.7 V·s/cm^2^. Collision energies were linearly interpolated between these two 1/K0 values.

### Sample acquisition DIA

Two microliters of tryptic peptides was loaded onto Evotip and analyzed using an Evosep One LC (EVOSEP) connected to a timsTOF Pro (Bruker). The Evosep One method was 60 SPD (21-min gradient, cycle time of 24 min) or 100 SPD (11.5-min gradient, cycle time of 14.4 min) and the mass spectrometer was operated in DIA-PASEF mode. The DIA-PASEF method consisted of eight 100 *m/z* windows. Two windows were acquired per 100-ms scan leading to a cycle time of 0.53 s. The other mass spectrometer parameters were set as follows: *m/z* range, 400 to 1201, the mobility (1/K0) range was set to 0.72 to 1.45 V⋅s/cm^2^, and the accumulation and ramp time were 100 ms.

### Sample search

Acquisition of the plasma samples led to 8+ TB of data, which were copied to a high-performance computing (HPC) system where we wrote a parallelization strategy to facilitate the swift identification and quantification of proteins in the raw data. This parallelization strategy enabled computational analysis in under 2 weeks (*8*). Briefly, all timsTOF data files were uploading to HPC where they were first rewritten to the Fragpipe mzBIN file format whereafter MSFragger (*3*, *16*) facilitated the peptide spectrum matching. Human protein sequences without isoforms were downloaded from UniProt where the protein sequences of the COVID-19 virus were added to the FASTA file, leading to a total number of 20,632 entries. Methionine oxidation and N-terminal acetylation were set as variable modifications and no fixed modifications were specified. A maximum of three modifications was allowed during the peptide spectrum matching. Next, a 1% false discovery rate was applied using the Philosopher tools (*17*). IonQuant (*18*) was used for quantification, which uses MS1 spectra to determine the relative quantification between samples. At least one ion was required for protein quantification and match-between runs was turned on.

The raw DIA data were processed using DIA-NN 1.8 (*19*) in the “robust LC (high precision)” mode with retention time (RT)-dependent normalization enabled. MS2 and MS1 mass accuracies were set to 10 parts per million.

### Statistical analysis

Statistical analyses were performed using R studio. Student’s *t* test was performed to evaluate the difference in abundance of the NP between the RS groups. A Fisher’s exact test was done to evaluate the observation frequency differences of the NP distribution across the RS.

## References

[1] N. L. Anderson, N. G. Anderson, The human plasma proteome: History, character, and diagnostic prospects. Mol. Cell. Proteomics 1, 845–867 (2002). 12488461 10.1074/mcp.r200007-mcp200

[2] COvid-19 Multi-omics Blood ATlas (COMBAT) Consortium, A blood atlas of COVID-19 defines hallmarks of disease severity and specificity. Cell 185, 916–938 e58 (2022). 35216673 10.1016/j.cell.2022.01.012 PMC8776501

[3] C. B. Messner, V. Demichev, D. Wendisch, L. Michalick, M. White, A. Freiwald, K. Textoris-Taube, S. I. Vernardis, A.-S. Egger, M. Kreidl, D. Ludwig, C. Kilian, F. Agostini, A. Zelezniak, C. Thibeault, M. Pfeiffer, S. Hippenstiel, A. Hocke, C. von Kalle, A. Campbell, C. Hayward, D. J. Porteous, R. E. Marioni, C. Langenberg, K. S. Lilley, W. M. Kuebler, M. Mülleder, C. Drosten, N. Suttorp, M. Witzenrath, F. Kurth, L. E. Sander, M. Ralser, Ultra-high-throughput clinical proteomics reveals classifiers of COVID-19 infection. Cell Syst. 11, 11–24 e4 (2020). 32619549 10.1016/j.cels.2020.05.012 PMC7264033

[4] R. J. Winzler, A. W. Devor, J. W. Mehl, I. M. Smyth, Studies on the mucoproteins of human plasma. I. Determination and isolation. J. Clin. Invest. 27, 609–616 (1948). 10.1172/JCI102006 PMC439531 16695579

[5] A. Zougman, J. R. Wisniewski, Beyond linker histones and high mobility group proteins: Global profiling of perchloric acid soluble proteins. J. Proteome Res. 5, 925–934 (2006). 16602700 10.1021/pr050415p

[6] P. R. Gajjala, H. Bruck, H. Noels, G. Heinze, F. Ceccarelli, A. Kribben, J. Saez-Rodriguez, N. Marx, W. Zidek, J. Jankowski, V. Jankowski, Novel plasma peptide markers involved in the pathology of CKD identified using mass spectrometric approach. J. Mol. Med. 97, 1451–1463 (2019). 31385015 10.1007/s00109-019-01823-8 PMC6746684

[7] A. Viodé, K. K. Smolen, B. Fatou, Z. Wurie, P. Van Zalm, M. K. Konde, B. M. Keita, R. A. Ablam, E. N. Fish, H. Steen, Plasma proteomic analysis distinguishes severity outcomes of human ebola virus disease. mBio 13, e0056722 (2022). 35446128 10.1128/mbio.00567-22 PMC9239184

[8] P. van Zalm, A. Viodé, K. Smolen, B. Fatou, A. N. Hayati, C. N. Schlaffner, O. Levy, J. Steen, H. Steen, A parallelization strategy for the time efficient analysis of thousands of LC/MS runs in high-performance computing environment. J. Proteome Res. 21, 2810–2814 (2022). 36201825 10.1021/acs.jproteome.2c00278 PMC9930095

[9] IMPACC Manuscript Writing Team; IMPACC Network Steering Committee, Immunophenotyping assessment in a COVID-19 cohort (IMPACC): A prospective longitudinal study. Sci. Immunol. 6, eabf3733 (2021). 34376480 10.1126/sciimmunol.abf3733 PMC8713959

[10] A. Ozonoff, J. Schaenman, N. D. Jayavelu, C. E. Milliren, C. S. Calfee, C. B. Cairns, M. Kraft, L. R. Baden, A. C. Shaw, F. Krammer, H. van Bakel, D. A. Esserman, S. Liu, A. F. Sesma, V. Simon, D. A. Hafler, R. R. Montgomery, S. H. Kleinstein, O. Levy, C. Bime, E. K. Haddad, D. J. Erle, B. Pulendran, K. C. Nadeau, M. M. Davis, C. L. Hough, W. B. Messer, N. I. A. Higuita, J. P. Metcalf, M. A. Atkinson, S. C. Brakenridge, D. Corry, F. Kheradmand, L. I. R. Ehrlich, E. Melamed, G. A. M. Comsey, R. Sekaly, J. Diray-Arce, B. Peters, A. D. Augustine, E. F. Reed, M. C. Altman, P. M. Becker, N. Rouphael; IMPACC study group members, Phenotypes of disease severity in a cohort of hospitalized COVID-19 patients: Results from the IMPACC study. EBioMedicine 83, 104208 (2022). 35952496 10.1016/j.ebiom.2022.104208 PMC9359694

[11] N. L. Anderson, The clinical plasma proteome: A survey of clinical assays for proteins in plasma and serum. Clin. Chem. 56, 177–185 (2010). 19884488 10.1373/clinchem.2009.126706

[12] V. Nanjappa, J. K. Thomas, A. Marimuthu, B. Muthusamy, A. Radhakrishnan, R. Sharma, A. A. Khan, L. Balakrishnan, N. A. Sahasrabuddhe, S. Kumar, B. N. Jhaveri, K. V. Sheth, R. K. Khatana, P. G. Shaw, S. M. Srikanth, P. P. Mathur, S. Shankar, D. Nagaraja, R. Christopher, S. Mathivanan, R. Raju, R. Sirdeshmukh, A. Chatterjee, R. J. Simpson, H. C. Harsha, A. Pandey, T. S. K. Prasad, Plasma proteome database as a resource for proteomics research: 2014 update. Nucleic Acids Res. 42, D959–D965 (2014). 24304897 10.1093/nar/gkt1251 PMC3965042

[13] B. Muthusamy, G. Hanumanthu, S. Suresh, B. Rekha, D. Srinivas, L. Karthick, B. M. Vrushabendra, S. Sharma, G. Mishra, P. Chatterjee, K. S. Mangala, H. N. Shivashankar, K. N. Chandrika, N. Deshpande, M. Suresh, N. Kannabiran, V. Niranjan, A. Nalli, T. S. K. Prasad, K. S. Arun, R. Reddy, S. Chandran, T. Jadhav, D. Julie, M. Mahesh, S. L. John, K. Palvankar, D. Sudhir, P. Bala, N. S. Rashmi, G. Vishnupriya, K. Dhar, S. Reshma, R. Chaerkady, T. K. B. Gandhi, H. C. Harsha, S. S. Mohan, K. S. Deshpande, M. Sarker, A. Pandey, Plasma Proteome Database as a resource for proteomics research. Proteomics 5, 3531–3536 (2005). 16041672 10.1002/pmic.200401335

[14] E. Brunet-Ratnasingham, S. P. Anand, P. Gantner, A. Dyachenko, G. Moquin-Beaudry, N. Brassard, G. Beaudoin-Bussières, A. Pagliuzza, R. Gasser, M. Benlarbi, F. Point, J. Prévost, A. Laumaea, J. Niessl, M. Nayrac, G. Sannier, C. Orban, M. Messier-Peet, G. Butler-Laporte, D. R. Morrison, S. Zhou, T. Nakanishi, M. Boutin, J. Descôteaux-Dinelle, G. Gendron-Lepage, G. Goyette, C. Bourassa, H. Medjahed, L. Laurent, R.-M. Rébillard, J. Richard, M. Dubé, R. Fromentin, N. Arbour, A. Prat, C. Larochelle, M. Durand, J. B. Richards, M. Chassé, M. Tétreault, N. Chomont, A. Finzi, D. E. Kaufmann, Integrated immunovirological profiling validates plasma SARS-CoV-2 RNA as an early predictor of COVID-19 mortality. Sci. Adv. 7, eabj5629 (2021). 34826237 10.1126/sciadv.abj5629 PMC8626074

[15] P. S. Arunachalam, F. Wimmers, C. K. P. Mok, R. A. P. M. Perera, M. Scott, T. Hagan, N. Sigal, Y. Feng, L. Bristow, O. T.-Y. Tsang, D. Wagh, J. Coller, K. L. Pellegrini, D. Kazmin, G. Alaaeddine, W. S. Leung, J. M. C. Chan, T. S. H. Chik, C. Y. C. Choi, C. Huerta, M. P. McCullough, H. Lv, E. Anderson, S. Edupuganti, A. A. Upadhyay, S. E. Bosinger, H. T. Maecker, P. Khatri, N. Rouphael, M. Peiris, B. Pulendran, Systems biological assessment of immunity to mild versus severe COVID-19 infection in humans. Science 369, 1210–1220 (2020). 32788292 10.1126/science.abc6261 PMC7665312

[16] A. T. Kong, F. V. Leprevost, D. M. Avtonomov, D. Mellacheruvu, A. I. Nesvizhskii, MSFragger: Ultrafast and comprehensive peptide identification in mass spectrometry-based proteomics. Nat. Methods 14, 513–520 (2017). 28394336 10.1038/nmeth.4256 PMC5409104

[17] F. da Veiga Leprevost, S. E. Haynes, D. M. Avtonomov, H. Y. Chang, A. K. Shanmugam, D. Mellacheruvu, A. T. Kong, A. I. Nesvizhskii, Philosopher: A versatile toolkit for shotgun proteomics data analysis. Nat. Methods 17, 869–870 (2020). 32669682 10.1038/s41592-020-0912-y PMC7509848

[18] F. Yu, S. E. Haynes, A. I. Nesvizhskii, IonQuant enables accurate and sensitive label-free quantification with FDR-controlled match-between-runs. Mol. Cell. Proteomics 20, 100077 (2021). 33813065 10.1016/j.mcpro.2021.100077 PMC8131922

[19] V. Demichev, C. B. Messner, S. I. Vernardis, K. S. Lilley, M. Ralser, DIA-NN: Neural networks and interference correction enable deep proteome coverage in high throughput. Nat. Methods 17, 41–44 (2020). 31768060 10.1038/s41592-019-0638-x PMC6949130

