# Supplementary material for: A simple, time- and cost-effective, high-throughput depletion strategy for deep plasma proteomics
Source: Sci Adv. 2023 Mar 29;9(13):eadf9717. doi: 10.1126/sciadv.adf9717 (PMC10058233; doi:10.1126/sciadv.adf9717)
Supplement: Supplementary file 1 — Legends for tables S1 to S6 [file sciadv.adf9717_sm.pdf]

Science Advances
AAAS

Supplementary Materials for

A simple, time- and cost-effective, high-throughput depletion strategy for deep plasma proteomics

Arthur Viode *et al.*

Corresponding author: Hanno Steen, hanno.steen@childrens.harvard.edu

Sci. Adv. **9**, eadf9717 (2023)
DOI: 10.1126/sciadv.adf9717

This PDF file includes:

Legends for tables S1 to S6

Other Supplementary Materials for this manuscript includes the following:

Tables S1 to S6

Supplementary Materials:

Table S1: Tissue enrichment results for the perchloric acid method

Table S2: Tissue enrichment results for the neat plasma method

Table S3: FDA biomarkers list

Table S4: NP peptides list

Table S5: Frequency of the Nucleoprotein for the respiratory status

Table S6: Fisher exact test results
